# Dietary Fatty Acids and Microbiota-Brain Communication in Neuropsychiatric Diseases

**DOI:** 10.3390/biom10010012

**Published:** 2019-12-19

**Authors:** Maria Cristina Marrone, Roberto Coccurello

**Affiliations:** 1 European Brain Research Institute (EBRI), Fondazione Rita Levi-Montalcini, 00161 Rome, Italy; m.cristinamarrone@gmail.com; 2 National Research Council (CNR), Institute for Complex System (ISC), 00185 Rome, Italy; 3 IRCCS–S. Lucia Foundation (FSL), 00143 Rome, Italy

**Keywords:** microbiota-brain communication, dietary fatty acids, polyunsaturated fatty acids, depression, schizophrenia

## Abstract

The gut-brain axis is a multimodal communication system along which immune, metabolic, autonomic, endocrine and enteric nervous signals can shape host physiology and determine liability, development and progression of a vast number of human diseases. Here, we broadly discussed the current knowledge about the either beneficial or deleterious impact of dietary fatty acids on microbiota-brain communication (MBC), and the multiple mechanisms by which different types of lipids can modify gut microbial ecosystem and contribute to the pathophysiology of major neuropsychiatric diseases (NPDs), such as schizophrenia (SCZ), depression and autism spectrum disorders (ASD).

## 1. Introduction

Dietary habits and composition of dietary patterns are the best known and most powerful modifiers of brain health. Even though this is a longstanding notion, it is only by the modern conception of gut-brain reciprocal communication that our vision of the possible mechanisms underlying the impact of nutrients on brain disorders has changed face. Here, we will focus on the gut-brain interface as possible link between dietary lipids, diet-derived fatty acids and altered lipid metabolism in the pathogenesis and susceptibility to neuropsychiatric disorders. In the last few years, an impressive body of literature has begun to decipher the key importance of the trillions of bacteria hosted in our gastrointestinal (GI) tract, and in particular to decode the dynamic interplay between the heterogenous composition of this huge community of microorganisms and susceptibility to very different illnesses such as obesity, type 2 diabetes, pain, neurodevelopmental, neurodegenerative and neuropsychiatric diseases [1,2,3,4]. An ecosystem made by trillions of commensals such as bacteria, archaea, protozoa and viruses, whose entire network is called microbiota. On the other hand, by microbiome is generally described the collective microbial genome of this ecosystem [5,6]. Although detailed in other studies [7,8], it should be always kept in mind that the bidirectional gut-brain dialogue takes place by the means of a complex communication network, including the sympathetic and parasympathetic branches of the autonomic nervous system (ANS), the hypothalamic-pituitary-adrenal axis (HPA) of the endocrine system, the immune system and the enteric nervous system (ENS). In parallel, the release of enteroendocrine hormones can considerably modify host physiology. Indeed, enteroendocrine cells secrete a number of different hormones such as glucagon-like peptide 1 (GLP-1), peptide-YY (PYY), cholecystokinin (CCK) and serotonin (5-hydroxytriptamine, 5-HT), with a key impact on nutrient absorption, metabolism and appetite [9,10], and also modulation of anxiety-like behaviors [11]. Thus, the gut-brain axis integrates hormonal, immune and neural signals in a communication system whereby the microbial community and its metabolites also influence the ENS, intestinal motility and permeability, mucosal immune function [12,13,14], and affect brain neurochemistry as well as processing of emotional and rewarding behaviors [8,15]. Within this complex system, bacterial metabolites such as short chain fatty acids (SCFAs) (e.g., butyrate or butyric acid (BA), acetate (AC) and propionate or propionic acid (PPA)), immune mediators (e.g., chemokines), enteroendocrine signals and bidirectional interaction via the vagus nerve are the main routes responsible of microbiota-to-brain communication (MBC). To further support the key role of the vagal pathway, both deleterious effects via lipopolysaccharides administration or beneficial outcomes of probiotics supplementation have been suppressed or blunted by the inactivation of vagal communication [14,16,17]. The afference of vagus nerve to the brain influences the HPA axis activity and the coordinated responses to physical and emotional stressors, as well as the secretion of hypothalamic corticotropin-releasing factor (CRF) and adrenocorticotropic hormone (ACTH) secretion from the pituitary gland [18,19].

(i) First, the present report is focused on the relationship between microbiota alteration and brain disorders.
(ii) Then, the study will engage with the association between some dietary lipids, alteration of microbiota-brain communication (MBC) and vulnerability to NPDs, such as SCZ, depression and ASD.
(iii) Not necessarily detrimental, the impact of selected dietary lipids will be also considered for its protective/preventive potential against the pathogenesis of NPDs. Among fatty acids (FAs), particular relevance will be assigned to dietary polyunsaturated FAs (PUFAs), their role in intestinal inflammation and the function of proresolving lipid mediators.
(iv) Next, considering that Western dietary (WD) pattern has taken over the global nutrition, a specific attention will be attributed to WD-induced chronic inflammatory conditions affecting intestine and brain physiology.
(v) Finally, dietary composition and gut bacteria metabolites will be assessed for their capacity to produce the particular class of short chain FAs (SCFAs), whose contribution to psychiatric illnesses has been lately a major focus of investigation.

Although a definition of the depression, SCZ and ASD is beyond the purpose of the study, these NPDs are those for which there is multiple evidence for a relationship between alterations of microbial gut community, MBC and liability to disease. Hence, according to the Diagnostic and Statistical Manual of Mental Disorders (DSM-5), a diagnosis of major depression requires the co-occurrence of five or more symptoms with “depressed mood” being the most accurate symptom to differentiate between non-depressed and moderately depressed subjects, and “anhedonia” to discriminate between moderate and severe depression [20,21,22]. Next, according to the DSM-5 [20], a diagnosis of SCZ requires the coexistence of two or more symptoms such as delusions, hallucinations, disorganized speech and negative symptoms (i.e., affective flattening). Finally, the diagnosis of ASD [20] has helped to consolidate the different subtypes of pervasive developmental disorders into an unitary ASD diagnosis, which requires the presence of social communication impairments and restricted repetitive behaviors [23].

## 2. Microbiota and Neuropsychiatric Diseases (NPDs)

The notion that the alterations of microbiota ecosystem may negatively affect brain physiology has been suggested by the observation that chronic antibiotic treatment is associated with an increased risk of psychiatric illness [24]. Moreover, the link between microbiota alteration and brain disease is corroborated by the comorbidity between psychiatric disorders and several GI disease such as irritable bowel syndrome (IBS) and enteropathies as well as by the efficacy of specific classes of probiotics (i.e., psychobiotics) on stress-induced GI symptoms, anxiety and depression [25,26,27]. Even more unexpected, is the demonstration that the pathophysiology of GI disorders or systemic inflammation can be spread between organisms by transferring the microbiota either from patients or pathological animal models to germ-free (GF) mice [28,29]. The impact of GI disorders on mental health [25] is supported by the large use of antidepressants among the population of IBS patients [30], which convincingly corroborate the role of emotional stress in dysbiosis, gut motility and epithelium integrity. It is indeed well known that prenatal, early postnatal, and adulthood stress play a pivotal role in the pathogenesis of different psychiatric illnesses [31]. Microbiome can directly affect stress response, and GF mice lacking of commensal microbiota are hyperresponsive to stress (i.e., restraint), showing excessive HPA activation characterized by CRF gene and protein overexpression, increased plasma corticosterone and ACTH, and decreased expression of hippocampal brain-derived neurotrophic factor (BDNF) [32]. Notably, these effects can be drastically attenuated by colonization of juvenile (but not adult) mice with the individual strain *Bifidobacterium infantis* or exacerbated by the association with enteropathogenic *Escherichia coli* [32]. Maladaptive responses in terms of decreased anxiety-like behaviors have been described in GF mice, as well as the normalization of dysfunctional risk-taking behavior following colonization during early development [1,33,34]. Quite surprisingly, brain development is thus drastically shaped by the microbiome; indeed, morphological alteration of neural dendrites have been detected in the hippocampus and amygdala of GF mice [34,35]. Corroborating the notion that gut microorganisms play a regulatory role not only in brain development but also in mature neurons, adult hippocampal neurogenesis has been described to be higher in GF than in conventional mice regardless of postweaning microbial colonization [36].

If the depletion of commensal microbiota increases the risk of maladaptive behaviors and can be fully reverted only within the maturational period, the strong influence of microbiota on neuron plasticity and circuitry wiring during the neurodevelopment may also increase the susceptibility to stress-induced psychiatric disorders. In any case, during development [37] and adulthood, probiotics supplementation may mitigate social stress-induced cognitive, behavioral (e.g., anxiety, depression) and immune alterations [27,38,39]. From this view, prototypical appears one animal study in which stress-induced hyperthermia, increase of corticosterone levels, anxiety- and depression-like behaviors were reduced by the chronic treatment with *Lactobacillus rhamnosus* (*JB-1*) probiotic [14,34]. The antidepressant and anxiolytic effects of *L. rhamnosus* (*JB-1*) treatment was mediated by the selective increase of gamma-aminobutyric acid GABA(B) receptors mRNA expression in cingulate cortex and decrease of GABA(A) mRNA expression in the prefrontal cortex (PFC), as well as by the concomitant decrease of GABA(B) expression in hippocampus and amygdala and increase of GABA(A) expression in the hippocampus [14]. All the changes induced by *L. rhamnosus* (*JB-1*) treatment in GABA brain expression were suppressed in vagotomized mice as well as significantly reduced were the antidepressant and anxiolytic effects [14]. Thus, brain neurochemical changes involving the GABAergic system are showed after probiotic treatment in several animal models, along with the importance of vagus nerve integrity for the preservation of the MBC and probiotic-associated neurochemicals effects. Indeed, the bidirectional microbiota-brain interaction entails neuroendocrine and neuroimmune signaling mechanisms (e.g., cytokines, CRF) as well as neuroactive agents [40,41] as key routes of communication by the way of HPA axis and vagus nerve.

Linked to the use of probiotics as antidepressant treatment, there is recent evidence that rats subjected to chronic unpredictable mild stress to induce depression-like behaviors had an increased rate of Firmicutes that correlated positively with colonic 5-HT and negatively with 5-HT in the prefrontal cortex, both alterations of 5-HT metabolism reverted by treatment with *Bifidobacterium longum and L. rhamnosus* [42]. An elegant demonstration of the impact of gut microbiota in depression is the alteration of microbiota diversity found in depressed patients, together with the possibility to use fecal transplantation to transfer this microbial “signature” of depression in microbiota-depleted animals and induce a depression-like phenotype [43]. Moreover, the depression-like phenotype observed in GF mice can be exacerbated by the transplantation of “depressed microbiota” from patients with major depressive disorder in microbiota-depleted mice [44]. Accordingly, minocycline-induced alteration of microbiota composition can attenuate the depression-like behaviors exacerbated in mice by the exposure to chronic restraint stress [45]. Interestingly, this study reports that chronic stress reduced *Bifidobacterium* species that inhibit the inflammation associated to the nuclear factor-κB pathway, while at the same time increased the incidence of *Lactobacillus* species that are involved in inflammasome activation via IL-1β secretion [45]. Recently, a 16S rRNA gene analysis coupled with a wide metagenomic sequencing study on a large cohort of depressed patients has linked different microbial taxa (i.e., enterotypes) with quality of life of participants and incidence of depression [46]. For instance, results showed that reduced microbial density of *Bacteroides* is associated with higher incidence of depression and reduced indicators of quality of life [46]. Considering GABA and dopamine (DA) as neuroactive products of microbial metabolism [14,40,41,47], this study further identified the DA metabolite 3,4-dihydroxyphenylacetic acid (DOPAC) as “gut-brain module” positively correlated with mental quality of life, while a tendency towards the association between increase of GABA synthesis and depression was also found [46]. 

## 3. Dietary Lipids and The Orchestration of Gut Microbial Community

### 3.1. Derangement of Microbial Ecosystem, Neuroinflammation and Liability to NPDs

**Figure 1:**
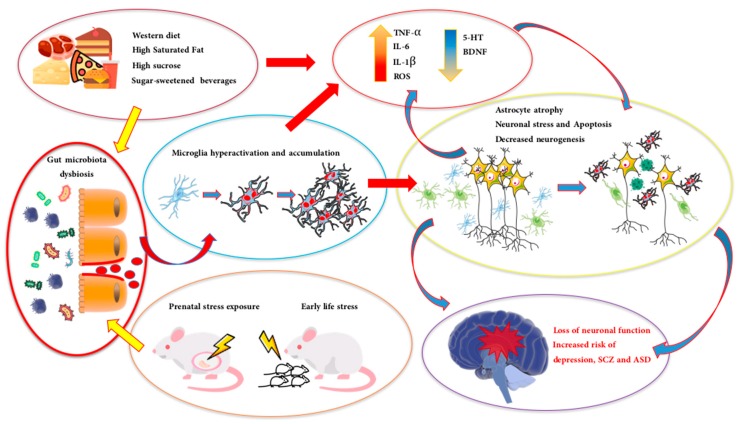
The figure depicts the main impact of different sources of environmental burden on the derangement of gut microbiota ecosystem, and a selection of potential mechanisms underlying dysbiosis-induced liability to neuropsychiatric diseases (NPDs). Here (left side), are depicted two recognized key pathogenetic factors such as: 1-(upper figure) the worldwide consumption of western diet characterized for instance by high saturated fat and high-sucrose foods, corn-derived fructose and carbonated beverages; 2-(lower side) multiple prenatal stress, maternal immune activation and early-life stressors. Chronic exposure to either one or both sources of environmental burden can determine systemic and brain inflammation and alteration of brain homeostasis via intestinal microbiota dysbiosis and severe immune changes such as shifting towards a persistent activation of the microglial phenotype, production of inflammatory cytokines, ROS and decrease of BDNF and 5-HT synthesis. In turn, the combination of neuronal, microglial and astrocyte damage (e.g., atrophy and reduced neurogenesis), altered synaptic and neural communication and brain inflammation contribute to the risk of depression, SCZ and ASD.

The impact of dysregulated MBC in the pathogenesis of NPDs can be analyzed at the light of systemic and brain inflammation and risk for the defense of brain homeostasis. Within this framework, it is of striking importance the function of surveillance operated by microglial cells through highly dynamic and plastic morphological changes. The microglial phenotype may shift (i.e., from “surveying” to “activated”) according to the alterations of neural activity, neuronal-microglial signals and synaptic communication (Figure 1) [48,49]. Being the major resident and immunocompetent cells of the brain, microglial cells are activated by tissue damage, infections as well as during the course of neuropsychiatric and neurodegenerative diseases [50,51].

Notably, there is recent evidence that maternal microbiota can drive the development and function of microglial offspring which, ultimately, depends on the integrity of maternal gut-brain crosstalk [48]. Several recognized risk factors in ASD and SCZ pathogenesis, such as generalized maternal immune activation and early-life stress, can induce not only neuroinflammation and abnormal microglial activation [52,53,54] but also alteration of host immunity and stability of resident bacteria community [55,56]. Evidence for neuroinflammation-associated microglial activation in SCZ and ASD patients has been also confirmed from positron emission tomography (PET) studies in which an increase of expression of the translocator protein (TSPO) (i.e., a marker of microglial activation) was found at hippocampal level [57,58]. The alterations of gut microbial community, secondary to prenatal and early-life environmental insults, can thus determine a state of severe immune changes, including the production of inflammatory cytokines and persistent microglia cells activation (e.g., Figure 1). From this view, unhealthy dietary patterns can be classified as early- or later-life environmental insults. It is known that unhealthy dietary patterns, such as the consumption of saturated fats, are directly linked to low-grade systemic inflammation, obesity and pro-inflammatory immune response (Figure 1) [59,60]. Moreover, there is recent evidence that microglial-driven neuroinflammatory signaling is a causal link between excessive consumption of high-fat diet (HFD) and hypothalamic gliosis, thus resulting a key player in HFD-induced brain inflammation and derangement of energy homeostasis [61].

If unhealthy dietary patterns are powerful determinants of the alteration of host microbial community and dysbiosis generates microglial hyperactivity (Figure 1), then the consumption of selected dietary lipids may considerably contribute to control microglia activation, brain inflammation and, ultimately, reduce the risk of NPDs.

### 3.2. Dietary Lipids: Fatty Acids, Alteration of Microbiota Diversity and NPDs

Basically, fatty acids (FAs) can be classified according to the number of double bonds in the side chain, from saturated FAs (SFAs) lacking of double bonds, to monounsaturated (MUFAs) with a single double bond, and polyunsaturated (PUFAs) showing two or more double bonds in the carbon chain [62]. Since FAs can also be categorized by the carbon chain length and the position of the first double bond on methyl terminal, then the whole family of PUFAs can be further classified by including the omega-3 PUFAs (*n*-3 PUFAs) and the omega-6 PUFAs (*n*-6 PUFAs) series. Both *n*-3 PUFAs and *n*-6 PUFAs are vital nutrients that because of the lack of specific enzymes (i.e., desaturases) they cannot be synthetized *de novo* by mammals [63]. For this reason, the consumption via dietary sources of the two 18 carbon (18C) essential fatty acids, linoleic acid (18:2*n*-6, LA) and α-linolenic acid (18:3*n*-3, ALA) is needed to generate the biologically-active *n*-6 PUFAs and *n*-3 PUFAs, respectively. According to the chain length, the best known *n*-3 PUFAs comprise the shorter chain precursor to the *n*-3 series ALA, the stearidonic acid (SDA, 18:4), the long chain (≥C_20_) eicosapentaenoic acid (EPA, 20:5) and the docosahexaenoic acid (DHA, 22:6). On the other hand, the family of *n*-6 PUFAs include the shorter chain precursor to the *n*-6 series LA, the arachidonic acid (ARA, 20:4), the gamma-linolenic-acid (GLA, 18:3) and the dihomo-gamma-linolenic acid (DGLA, 20:3) [64].

In the last few years, an impressive number of evidence has highlighted the pro- and the anti-inflammatory potential provided by the two series of *n*-6 and *n*-3 PUFAs, respectively. Considering the detrimental impact produced by WD on microbial ecosystem, the excessive consumption of *n*-6 PUFAs-enriched vegetable oils (e.g., from soybean, corn, sunflower and margarines) and red meat, as main sources of LA and ARA, is the most important contributing factor to the large increase of the dietary *n*-6 to *n*-3 ratio [65,66]. Essentially, the pro-inflammatory chronic response promoted by *n*-6 PUFAs is linked to ARA-derived signaling pathway, generating bioactive lipid known as eicosanoids and isoprostanes [67], which are involved in atherogenic processes, abnormal cell proliferation (e.g., cancer), obesity and irritable bowel disease (IBD) [68,69]. The eicosanoids family include prostaglandins (PGs), prostacyclins, thromboxanes (TXs), lipoxins (LXs) and leukotrienes (LTs), having different roles in cytokine synthesis and amplification or reduction of inflammation [70]. By contrast, *n*-3 PUFAs control inflammation mainly through the precursor ALA and then via EPA and DHA production. Indeed, EPA and DHA are competitive substrates for *n*-6 PUFAs metabolism and ARA-derived pro-inflammatory eicosanoids. Interestingly, a recent metabolomic study on a cohort of SCZ patients has described abnormally elevated serum levels of SFAs, MUFAs and *n*-6 PUFAs as possible consequence of higher than normal desaturation from SFAs to MUFAs and inadequate brain energy supply [71].

Recent attention has been focused on the mechanisms of inflammation resolution and *n*-3 PUFAs-derived lipids named “specialized pro-resolving mediators” (SPMs), comprising different members of signalling molecules such as lipoxins, resolvins, protectins and maresins [72]. Deficits of *n*-3 PUFAs have been repeatedly reported in patients with SCZ, bipolar disorder and depression, and there is evidence that EPA and DHA supplementation may be beneficial in a subgroup of ASD patients [73,74]. Recently, in a longitudinal 7-years study, the increase of n-6:n-3 ratio at baseline as determined in a cohort of young individuals with “ultra-high risk” for depression was found to be a valid and accurate predictor of likelihood to develop later mood disorders [75].

**Figure 2:**
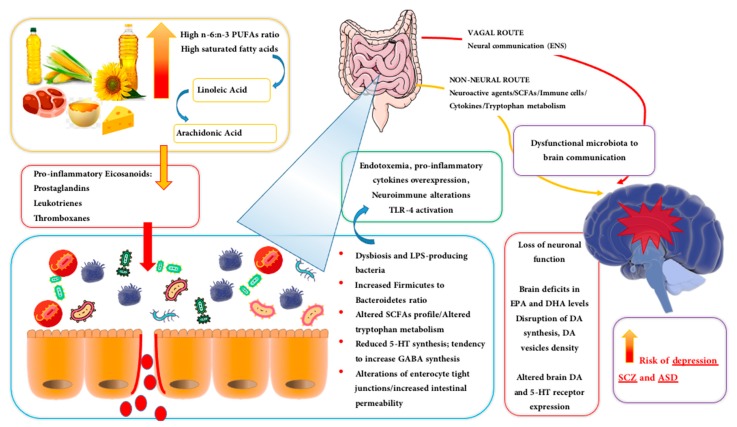
The figure sketches the current knowledge and the potential relationship between consumption of n-6 PUFAs- and SFAs-rich diets, production of pro-inflammatory eicosanoids mediators, derangement of microbial ecosystem and increased liability to neuropsychiatric diseases (NPDs). The prevalent ingestion of dietary n-6 PUFAs (and SFAs) is linked to the drastic alterations of microbiota diversity, inflamed microenvironment, overgrowth of harmful bacterial species (e.g., *Enterobactericeae*), metabolic endotoxemia (increased plasma endotoxins, such as LPS) and increased intestinal permeability. Besides the upregulation of cyclooxygenases- and lipoxygenases-dependent synthesis of eicosanoids, other mechanisms may contribute to dietary n-6 PUFAs/SFAs-induced dysbiosis, such as: 1) increased expression of NF-κB signaling pathway and induction of pro-inflammatory cytokines and 2) decreased synthesis of “specialized pro-resolving mediators” (SPMs) including the resolvins (RVs) series E (RvE) and D (RvD). The overall picture of systemic metabolic endotoxemia triggers immune dysregulation and recognition of pathogen-associated molecular patterns via toll-like receptors (TLRs) and in particular TLR4-dependent synthesis of pro-inflammatory cytokines (e.g., TNF-α, IL-1β, IL-6, and IL-12). In turn, reduced 5-HT synthesis, altered tryptophan metabolism and SCFAs balance contribute to dysfunctional microbiota-to-brain-communication. The reported deficits in plasma and brain EPA/DHA levels may further contribute to the disruption of DA and 5-HT function and, ultimately, to increased risk of depression, SCZ and.

Although accumulating studies have explored the relationship between dietary supplementation with *n*-3 PUFAs and NPDs, the impact on microbiota, symptoms and severity of patients with major depressive disorder, ASD or SCZ is still poorly understood. By using transgenic mice able to overproduce *n*-6 PUFAs and to increase the *n*-6 to *n*-3 ratio, it has been possible to show the development of several pathogenetic cascades involving, among others, metabolic endotoxemia, fatty liver and cancer [76]. Moreover, not only these mice exhibited chronic inflammation (e.g., high serum LPS, intestinal permeability and TNF-α, IL-1β and IL-6 overexpression), but also the analysis of fecal samples revealed the higher abundance of *Enterobacteriacea* bacteria, with increased *Proteobacteria* while reduced *Bacteroides* and *Actinobacteria* phylum (e.g., Figure 2) [76]. The presence of many markers of gut dysbiosis and intestinal permeability in fecal samples such as higher levels of 1-methylnicotinamide, cysteine, histidine and spermidine corroborate the possible causal relationship between elevated *n*-6 PUFAs tissue content, abnormal changes in gut microbiota and disease development [76]. Similar results were previously reported in mice fed with high *n*-6 PUFAs diet [77]. The same study identified in the secretion of intestinal alkaline phosphatase (IAP) the main mechanism by which the transgenic enhancement of *n*-3 PUFAs tissue content can provide an anti-inflammatory potential, stimulate the growth of *Bifidobacterium*, reduce LPS levels, gut permeability and metabolic endotoxemia [77].

On the other hand, indirect positive effects of *n*-3 PUFAs dietary supplementation on the risk to develop chronic depressive symptoms have been recently described [78]. Here, a randomized, double-blind and stratified study of the impact of family violence on child behavior, reported that *n*-3 PUFAs nutritional intervention in children reduced the level of psychological aggression among adult caregivers [78]. Exposure to *n*-3 PUFAs-enriched diet during the gestation can build a specific maternal *n*-3 PUFAs *environment* that, in turn, can “prime” offspring microbial composition in early life and confer protection during adulthood. Indeed, endogenous production of *n*-3 PUFAs during the gestational period has been shown to shape offspring gut microbiota and protect the progeny against HFD-induced metabolic alterations [79]. Although few studies have investigated the relationship between *n*-3 PUFAs supplementation, modification of MBC and early-life stress, there is evidence that long-term EPA/DHA supplementation can restore the microbiota composition in maternally-separated rats [80]. On this basis, there is an important implication for *n*-3 PUFAs supplementation to protect against stress-induced susceptibility to mood disorders. According to a meta-analysis of the biological status of *n*-3 PUFAs in mood disorder, plasma and brain EPA and DHA levels were found reduced in in patients with depression [81]. Interestingly, despite the inverse association between dietary fish consumption and depression incidence, and the positive association between increase of eicosanoids production, depression and SCZ [82], the causal relationship between *n*-3 PUFAs supplementation, *Firmicutes* to *Bacteroidetes* ratio and antidepressant effects is still poorly understood. As observed for depression, plasma EPA and DHA levels are found decreased in ASD children [83,84]. Moreover, in a placebo-controlled study, plasma BDNF levels were increased by EPA plus DHA dietary supplementation in first-episode psychotic patients and were found to inversely correlate with depressive symptoms [85]. Rats fed with a *n*-3 PUFAs-deprived diet showed reduced levels of BDNF expression in the prefrontal cortex, an area of pivotal importance for the pathophysiology of depression, SCZ and ASD [86].

The possible mechanistic link between deficiency of *n*-3 PUFAs reservoir and risk of NPDs may be also identified in the neuroinflammatory pathways and in the neuroimmune alterations involved in depression or SCZ pathogenesis. Notably, a potent anti-inflammatory and protective activity via macrophages stimulation and inhibition of NLRP3 inflammasome activation and IL-1β secretion was demonstrated in mice fed with *n*-3 PUFAs-enriched diet [87]. As crucial component of the innate immune response there is the activation of toll-like receptors (TLRs), which are a family of transmembrane proteins, largely expressed not only on immune cells (e.g., macrophages) but also on the cells of the intestinal epithelium (i.e., enterocytes) where these receptors are involved in the prevention of systemic low-grade inflammation and gut microbiota colonization, for instance by sensing polysaccharide A on *Bacteroides fragilis* [88,89]. TLRs recognize the so-called pathogen-associated molecular patterns (PAMPs) to prevent propagation of inflammation. Controlling the immunological responses, TLRs can inhibit the activation of pro-inflammatory cytokines or NFκB-mediated inflammatory program and preserve the intestinal homeostasis by reducing the entry of bacterial products to cytosolic inflammasome [90]. As opportunely highlighted [91], TLRs are essential components of the gut immune system capable to regulate intestinal homeostasis, thus playing a key role either for resilience or susceptibility to specific conditions where gut microbiota dysbiosis is prevalent such as IBD. The same study also report that deregulation of TLRs activity is associated not only with metabolic impairment (e.g., diabetes) but also with several brain pathologies and neuroinflammation characterizing neurodegenerative diseases [91].

LPS-producing Gram-negative bacteria activates the TLR4 subtype [91,92], triggering the synthesis of several pro-inflammatory mediators (e.g., TNF-α, IL-1β and IL-6) and a cascade of pathogenetic inflammatory events. From this view, the perturbation of microbial community, as reported in SCZ, can trigger immune or neuroimmune alterations that have been described in SCZ patients in terms of astroglial and microglial activation, impaired neurogenesis and changes of glutamate transmissions and NMDA receptor subunits [93,94,95]. Thus, dietary changes and modifications of microbiota diversity may disrupt sensitivity of TLRs activation and produce a state of multiple neuroimmune alterations, increasing the risk of neurodevelopmental disorders such as SCZ and ASD. The consumption of dietary fats can either increase or attenuate LPS levels and TLR4-associated inflammatory signaling, depending on the type of dietary fats. Indeed, SFAs such as lauric and palmitic acids can activate TLRs-mediated inflammatory program [96,97]. An interesting interplay does exist between the prevalent consumption of specific dietary lipids, LPS plasma levels and risk of endotoxemia. In healthy elderly subjects, the consumption of a carbohydrate-based/PUFAs-enriched diet has been shown to be associated to lower fasting LPS plasma levels, while the ingestion of diets rich either of SFAs or MUFAs resulted in higher fasting endotoxemia [98]. The hierarchy of the impact on LPS plasma levels is better clarified by considering these results at the light of a previous study in which the consumption of a Mediterranean-like diet (e.g., MUFAs-enriched) decreases the postprandial pro-inflammatory response more than the consumption of PUFA diet and much more than SFA-based diet [99]. Indeed, the mixed *n*-3 PUFAs/*n*-6 PUFAs composition may increase the pro-inflammatory potential just above the level of MUFAs-based diet but, in any case, below that provided by consumption of a SFAs-rich diet. Several investigations have underlined the reciprocal regulatory action exerted by SFAs and *n*-3 PUFAs on the activation of TLR4 and TLR2 subtype [97]. Not only SFAs activate, while *n*-3 PUFAs and particularly DHA, deactivate TLR4- and TLR2-associated inflammatory program, but SFAs can induce dimerization of TLR4 and TLR2 and subsequent translocation of these receptors into lipid raft of plasma membranes thus facilitating the downstream signaling, which is conversely inhibited by DHA [97].

Undoubtedly, the intricate connection involving dietary fats, endotoxemia, alterations of microbiome and NPDs eludes the possibility to grasp the *primum movens* of the chain’s events. Nonetheless, the detrimental impact induced by HFD on proteins expression/distribution of the enterocyte tight junctions [100,101] supports the view that ingestion of some dietary fats is a primary trigger leading to intestine permeability and increased susceptibility to NPDs. An important factor linking dietary fats to intestinal barrier function and intestine permeability is the susceptibility of dietary lipids to increase bile acid secretion, and the relationship between bile acid-mediated signaling, toxicity and alterations of enterocyte tight junctions proteins [102,103]. The harmful effects of SFAs on the integrity of intestinal barrier overwhelm that produced by the consumption of *n*-6 PUFAs-enriched HFD. Indeed, animals fed with SFAs-enriched HFD display decreased barrier integrity and infiltration of inflammatory immune cells (e.g., neutrophils), which are not detected in *n*-6 PUFAs-enriched, or *n*-3 PUFAs-enriched, HFD [104]. Accordingly, dietary *n*-3 PUFAs has been shown to mitigate experimental colitis [105], and EPA to provide protection against inflammation-induced dysfunction of permeability of intestinal epithelial barrier (“leaky gut”) [106].

### 3.3. Proresolving Lipid Mediators, Intestinal Inflammation and NPDs

Both pro- or anti-inflammatory bioactive lipid metabolites are produced via the enzymatic oxidation orchestrated by cyclooxygenases (COXs), lipoxygenases (LOXs) and cytochrome P450 (CYP450) monooxygenases. In particular, as above noted (paragraph 3.2), from the AA the COX pathway yields PGs and TXs, while the LOX pathway generates LTs and LXs [107,108]. On the other hand, dietary *n*-3 PUFAs can provide adequate EPA and DHA plasma and brain levels, which are LOX and CYP substrates and are steadily associated with a potent anti-inflammatory action opposing both the expression of cytokines such as TNF-α, IL-6, IL-1β, and inflammatory stimuli such as LPS [109]. In this view, *n*-3 PUFAs are bioactive lipid mediators able to promote the resolution of inflammation via the biosynthesis of EPA- and DHA-derived “specialized pro-resolving mediators” (SPMs) [72]. These EPA- and DHA-derived lipid metabolites are anti-inflammatory and “pro-resolving” members of the oxylipins family, which include resolvins (RVs), protectins (PDs), eicosanoid and maresins (MaRs) [110]. The resolvin series are the major EPA- and DHA-derived SPMs, and precisely, resolvin E (RvE) and resolvin D (RvD) series if derived from EPA and DHA, respectively [111]. Despite the lack of evidence for a direct mechanistic modulation exerted by RvE or RvD on the microbiota landscape, there is substantial empirical proof that *n*-3 PUFAs provide antimicrobial activity, and that conditions characterized by chronic low-grade inflammation and epithelial damage (e.g., IBD, ulcerative colitis and CD) can be completely or partly relieved by resolvins-mediated attenuation of intestinal inflammation [112,113,114]. Moreover, DHA-derived RvD1 and RvD2 act through the binding to selected GPCRs, such as GPR32 (DRV1) and GPR18 (DRV2), respectively, while EPA-derived RvE1 through the binding to chemokine receptor-like 1, ChemR23 (ERV1) [115,116,117]. Notably, exogenous administration of high dose RvD1 not only promotes transepithelial resistance in SFAs-enriched HFD fed mice [104], thus improving gut inflammation, but also eradicates dihydrogen sulfide (H_2_S)-producing bacteria and particularly SFAs-associated enlargement of *Desulfovibrio* species. Concerning NPDs, a couple of recent studies have provided evidence for antidepressant efficacy achieved by intracerebroventricular (i.c.v.) infusion of RvD1 and RvD2 [118], or EPA-derived RvE3 [119], in a mouse model of LPS-induced depression-like behavior. In agreement, antidepressant-like effects were also described following i.c.v., PFC or hippocampal RvE1/RvE2 infusion, possibly via ChemR23 binding [120].

From this picture it emerges that, upon dietary *n*-3 PUFAs consumption or nutritional supplementation, EPA- and DHA-derived RVs, PDs and MaRs exert a concerted action with multiple immunomodulatory effects, affecting microbiota population, integrity of the intestinal epithelium, resolution of intestinal inflammation and response of resident immune cells. Despite several evidence corroborate the point that dietary *n*-3 PUFAs consumption can either prevent or ameliorate both NPDs and gut dysbiosis, the role of EPA- and DHA-derived RVs, PDs and MaRs in the shape of microbiome and immune system modulation is still partly understood. Indeed, elegant studies have described the composite depressive phenotype, the increased *Firmicutes* to *Bacteroidetes* ratio and the LPS responsiveness induced by *n*-3 PUFAs nutritional deficiency in pregnant female and male offspring [121], or the preventive effects induced by dietary *n*-3 PUFAs intervention on depressive-like behaviors and changes in microbiota composition induced by social instability during brain development [122]. Nevertheless, a step further in the understanding of the relationship between dietary lipids, alterations of microbial ecosystem and liability to NPDs would be the investigation of RVs-, PDs- and MaRs-associated signaling at the light of their role in the inflammatory program, including the inhibition of pro-inflammatory mediators, the block of neutrophil recruitment/infiltration, monocytes activation, regulation of polymorphonuclear neutrophils (PMN) apoptosis, bacteria clearance, stimulation of macrophages phagocytosis and promotion of chemokine scavenging [72,110].

### 3.4. n-3 PUFAs, Serotonin, Dopamine and NPDs

Since *n*-3 PUFAs-derivatives RVs, PDs, MaRs are involved in neuroprotection while *n*-6 PUFAs-derivatives eicosanoids (i.e., PGs, prostacyclins, TXs, LXs and LTs) are relevant for the pathogenesis of NPDs such as SCZ [123], it is of interest to ascertain whether abnormalities of phospholipids turnover is present in patients with SCZ. At first sight, it should be noted that COX-2 inhibitor celecoxib provides beneficial effects in patients with SCZ [124], and that eicosanoids may enhance DAergic neurotransmission and are involved not only in SCZ but also in refractory depression and ASD [125,126,127]. Within this context, the prevalent intake of *n*-6 PUFAs and the very high levels of ARA present in the WD, is able to abnormally increase the levels of PGs, TXs and LXs, and upregulate systemic and brain expression of pro-inflammatory enzymes (e.g., phospholipase A2, COX-2) and genes (e.g., TNF-α, IL-1β). WD-induced suboptimal *n*-6 to *n*-3 ratio foster dysbiosis of gut microbiota, inducing a derangement in the ability of immune system to fight inflammation and maintain intestinal homeostasis. The inverse correlation between decreased peripheral and brain DHA levels and severity of SCZ symptoms [128,129] supports the possible mechanistic contribution of dietary *n*-3 PUFAs and SCZ neuropathology [130,131]. In pre-clinical dietary *n*-3 PUFAs deficiency, an important decrease of DHA brain content was shown to alter DA function in a manner comparable to that reported in SCZ patients [132,133]. Likewise, in a pre-clinical model of amphetamine-induced SCZ-like behavior, *n*-3 PUFAs dietary supplementation reduced behavioral deficits, cytokine release and enhanced the effects of combined antipsychotic and celecoxib drug treatment [134]. Since brain DHA deficiency may alter the expression of DA receptors in ventral striatum and contribute to hypofunctioning of mesolimbic DA system and anhedonia as observed in depression [22,135,136,137], then prenatal or early postnatal deficits of brain *n*-3 PUFAs status may be a pivotal factor in depression pathogenesis.

As described (see paragraph 2), neurotransmitters relevant to NPDs such as DA and 5-HT are involved in the preservation of microbial ecology and are essential for bidirectional MBC [10,41,47], and both DA and monoamines are considered key players in the pathogenesis of SCZ and depression [138]. Notably, there is evidence that different types of dietary fatty acids may have distinct effects on 5-HT neurotransmission [139]. Indeed, 5-HT_2A_ and 5-HT_2C_ receptor binding was reduced in the mammillary nucleus (i.e., the inferior surface of the hypothalamic region) of rats fed with a SFAs-rich diet, while the consumption of a *n*-6 PUFAs-rich diet reduced 5-HT_2A_ receptor binding in the mammillary nucleus, 5-HT_2C_ receptor binding in the prefrontal cortex and 5-HT transporters (5-HTT) [139]. The study further highlights the important notion that the major effects on brain 5-HT function (both receptor binding and transporter) were induced by the consumption of a *n*-6 PUFAs-rich diet with relevant implications for NPDs. Concerning DA, this is further confirmed by the high levels of tyrosine hydroxylase found in the small intestine [140]. Moreover, antibiotics-induced gut microbiota depletion decreases intestinal DA synthesis in mice [141] and GF mice show unequal brain mRNA expression of DA D1 receptor (D1R), being upregulated within the hippocampus and reduced at dorsal and ventral striatum level [1]. Hence, DA metabolism is drastically affected by the alteration of microbiota ecosystem. An unbalance between DA and its metabolites such as homovanillic acid (HVA) and DOPAC has been described in GF rats and mice together with reduced DA and 5-HT turnover [142,143]. Notably, a decrease of HVA/DA ratio indicative of reduced DA turnover was observed in GF rats [143] as well as in the CSF of patients with major depressive episode [144]. Moreover, antibiotic-induced dysbiosis increase levels of L-3,4- dihydroxyphenylalanine (L-DOPA) in PFC and hippocampus and both L-DOPA and HVA in the amygdala [145,146], providing additional demonstration that brain DA content, turnover and metabolism are linked to the alteration of microbial ecology. The clinical use of atypical antipsychotics (AAPs) may elicit symptoms remission but also alter fecal microbiota composition in patients with SCZ [147], thus demonstrating that AAPs treatment is associated to selected changes of intestinal bacteria population that may explain the different clinical efficacy and the severe AAP-associated dysmetabolic side effects [148]. Metabolic disruption in the offspring and susceptibility to metabolic disease (e.g., obesity, type 2 diabetes) is modelled during the gestational period with the key contribution of the tight interplay between *n*-3 PUFAs dietary lipids and gut microbial environment. While maternal *n*-3 PUFAs status, including gestation and lactation period, can deeply rearrange offspring gut microbiota in mice and confer long-term protection to the progeny, the reduction of dietary *n*-3 PUFAs can deplete the amount of species involved in gut homeostasis such as *Akkermansia muciniphilia* [79]. Accordingly, in a similar mouse model of gestational deprivation of dietary lipids it was shown the detrimental impact of *n*-3 PUFAs deficiency on the microbial production of short-chain fatty acids (SCFAs) [149]. Although deficits of SCFAs have been mostly associated to the risk of IBD and metabolic disease [150], there is mounting evidence that microbial metabolism-derived SCFAs are key players in NPDs pathogenesis (as showed in the next section).

Considering the detrimental impact of *n*-3 PUFAs dietary deficiency on striatonigral and mesocorticolimbic DAergic neurons and BDNF expression [151,152], as well as the pivotal role of DA neurotransmission in the pathogenesis of NPDs [138], particular attention should be focused in the future on dietary lipids-associated mechanisms involved in the gut production of catecholamines and modulation of ENS.

## 4. Short Chain Fatty Acids and Microbiota Community: Implications for NPDs

Within the context of specific dietary nutrients as determinants of the alterations of microbial communities and increased liability to NPDs, a special attention should be focused on a particular class of lipids derived from microbial metabolism and made of SCFAs. The processing of dietary nutrients by microbial metabolism generates a complex signaling system principally composed of SCFAs, *L*-tryptophan (Trp) metabolites and neuroactive agents [47,153,154]. Trp metabolism and neuroactive agents are also of key importance for the understanding of the relationship between MBC and risk of NPDs. The neurochemical signals intrinsically produced by the gut microbiota such as DA, gamma-aminobutyric acid (GABA), 5-HT, acetylcholine, histamine, melatonin and noradrenaline [40,41,155,156] are key elements to understand the mechanisms by which the MBC can shape dysfunctional behaviors such as depression, anxiety and ASD. As precursor of both peripheral and brain-produced 5-HT, the amino acid Trp is converted by the tryptophan hydroxylase (TPH) enzyme to 5-hydroxytryptophan (5-HTP), and then 5-HTP to 5-HT by the aromatic L-amino acid decarboxylase [157]. Hence, Trp metabolism is required for central 5-HT synthesis and serotonin neurotransmission in both central nervous system (CNS) and in the ENS of the gut wall [157,158]. As consequence, dietary-induced changes of microbial metabolism may have a causal impact in the pathogenesis of NPDs by the way of the alteration of SCFAs, Trp metabolism and neuroactive agents. Here, a major attention is focused on the relationship between unhealthy diet, dysregulation of SCFAs production and possible mechanistic connection to depression, ASD and SCZ.

### 4.1. Western Diet and Derangement of Microbial Ecosystem

The more elegant demonstration of the powerful impact of dietary habits on microbial composition [159] is probably related to the consequences that different diets might produce on host physiology. By the global shifting to WD habits, most of the population of industrialized and developing countries has converted their dietary lifestyles to the massive consumption of high-fat, high-sucrose, and ultra-processed food items. Undeniably, WD is on the defendant bench for the possible causal link between large consumption and the overall incidence of obesity, colorectal cancer and chronic inflammatory conditions affecting the intestine such as Crohn’s Disease (CD) and ulcerative colitis, which are part of the so-called IBDs [160,161,162,163]. WD is very rich in saturated fats, refined grains, sucrose, corn-derived fructose, proteins from high processed red meats, salt, alcohol, sweetened and carbonated beverages [164,165], and its consumption is associated with dysbiosis and derangement of microbial composition [166] (Figure 1).

Hence, WD consumption deranges the symbiotic relationship between microbiota community and gut mucosa impairing host metabolism, immunity and protection against pathogens. Interestingly, microbiota dysmetabolism has been found to be accountable for the link between consumption of red meat and coronary heart disease via dietary phosphatidylcholine and production of the pro-atherosclerotic metabolite trimethylamine-N-oxide [167]. Together with the multiple evidence for the association between WD and dysbiosis, WD is also considered responsible for intestinal permeability and endotoxemia, as established for CD [168]. Bacterial commensals of the *Firmicutes* phylum with known immunomodulatory and anti-inflammatory potential such as *Faecalibacterium prausnitzii* (*F. prausnitzii*) are decreased in patients with CD, while its administration as probiotic is considered a therapeutic strategy for CD treatment [169]. The most impressive consequence associated with WD consumption is the decrease in both microbial diversity and protective bacteria, with the expansion of pro-inflammatory and invasive Proteobacteria (e.g., *Escherichia coli*) and drastically reduced production of SCFAs [170]. The increase of pathogens and mucin-degrading bacteria, as for instance for the *Mollicutes* class of the *Firmicutes* phyla, including *Clostridia* group and *Proteobacteria*, is thought to underlie the reduction of the *Bacteroides* phyla, thus mining the microbial diversity [171,172]. Since reduction in SCFAs-producing bacteria is a key factor in dysbiosis, gut mucosal inflammation and loss of intestinal barrier integrity, it becomes of great importance to understand how dietary patterns modulate the production of the major gut bacteria metabolites.

### 4.2. Dietary Composition and Gut Bacteria Metabolites: The Role of SCFAs

SCFAs are major gut bacteria metabolites. Without gut bacteria it would be impossible to break down non-digestible dietary nutrients, especially plant-derived dietary fibers. These complex carbohydrates consist of so-called resistant starch, oligosaccharides and non-starch polysaccharides, which are used by gut bacteria as energy substrate to produce, by fermentation, SCFAs and in particular acetate (C-2), propionate (C-3), butyrate (C-4), and also lactate [154]. Eating fermentable, non-digestible, carbohydrates produces several well-described beneficial effects, ranging from the reduced incidence of IBDs and cardiovascular diseases to the decreased risk of colorectal cancer and alleviation of type 2 diabetes [150,173,174,175]. Although the benefits provided by SCFAs have been identified in multiple mechanisms (e.g., histone deacetylase (HDAC) inhibition), SCFAs should be considered immunoregulatory metabolites, in particular of the regulatory T cells (T_regs_) [176], and key players in the communication between gut and immune system. In this view, SCFAs contribute to immunosurveillance by their binding to metabolite-sensing G-protein coupled receptors (GPCRs) such as GPR41, GPR43 and GPR109A, which are densely expressed on immune cells [176,177]. For instance, butyrate can act as immune-messenger by its capacity to stimulate T cells to produce IL-10 by GPR109A activation, thus suppressing carcinogenesis and generating anti-inflammatory effects (i.e., colon inflammation) [178]. Such second-messenger activity of SCFAs also encompass the regulation of gene expression, the improvement of glucose metabolism, cholesterol synthesis as well as gut secretion of hormones such as PYY and glucagon-like peptide 1 (GLP-1) [179,180,181,182]. Fermentation of non-digestible carbohydrates and SCFAs production is also involved in the control of brain function, given that butyrate can exert neuroprotective effects and improve cognitive function [183], and propionate reduce the activation of brain regions (i.e., caudate and nucleus accumbens) involved in reward processing in healthy subjects asked to observe pictures of palatable food items [184]. Emblematic of the action exerted by some bacterial species and their relationship with dietary patterns is the role played in immune homeostasis and gut health by the above-mentioned *F. prausnitzii*. Its ability to colonize human intestine correlates well with the consumption of dietary fibers [185], and the expansion of *F. prausnitzii* is also of key importance for its capacity to produce butyrate [186], whose implication in different neurological and psychiatric disorders is under constant surveillance.

#### 4.2.1. SCFAs and Depression

Extensive investigation of microbial dysbiosis and alteration of bacterial composition in patients with depression has revealed the existence of a main shift towards the increase of *Bacteroidetes* and *Proteobacteria* phyla, and a lower than in healthy subjects proportion of the phylum of *Firmicutes* including their *Lachnospiraceae* and *Ruminococcaceae* families, which play a key role for SCFAs production [187,188]. *Faecalibacterium* presence shows the tendency towards the reduction depending on severity of depressive symptoms as well as the abundance of *Enterobacteriaceae* increases in depressed patients [188]. Butyrate production is the most interesting link between diet, SCFAs and psychiatric disorders. Indeed, despite its organic nature, butyrate is able to potently inhibit classes I and IIa HDAC activity [189], and inhibition of histone acetylation has been shown to counteract depression-like behavior in preclinical animal models [190]. The findings focused on the changes of gene transcription by the alteration of chromatin structure via histone modifications and DNA methylation have demonstrated that epigenetics mechanisms and chromatin remodeling can provide promising alternative options to conventional antidepressant therapy such as selective serotonin reuptake inhibitors (SSRIs), serotonin noradrenaline reuptake inhibitors (SNRIs), tricyclic antidepressants or monoamine amine oxidase (MAO) inhibitors [191]. Basically, the study of chromatin remodeling has helped to uncover the mechanisms by which environment (including diet, stress and drug of abuse) can produce changes in gene expression. Thus, histone acetylation is promoted by histone acetyltransferases (HATs) and linked to increased accessibility to transcription machinery and gene expression, whereas reduced transcription and gene repression are induced by the lack of histone acetylation and HDAC-induced increase of ionic interaction between histones and DNA, highly condensed chromatin and densely packed DNA [192]. Notably, valproic acid (VA) is both a well-known mood stabilizer with neuroprotective and antidepressant potential [193] and a SCFA with HDAC inhibitory activity [189]. By impeding the removal of acetyl groups from histone proteins, the HDAC inhibitors preclude the decrease of histone acetylation thus activating gene transcription. HDAC inhibitors seem to imping on the same neurotrophic factors considered to be involved in neuroplasticity and depressive disorder, and HDAC downregulation has been associated to the efficacy of antidepressant treatment (e.g., imipramine) in the social defeat stress model of depression [194]. In particular, antidepressant treatment can abolish social defeat-induced BDNF downregulation within the hippocampus and PFC [195], and HDAC inhibitors such as VA and sodium butyrate can upregulate BDNF expression and protect midbrain dopaminergic (DAergic) neurons [196]. After oral administration, butyrate can cross the blood brain barrier (BBB) and act in the brain as HDAC inhibitor, as demonstrated by the increase of neuronal histone acetylation and stimulation of neurogenesis [197,198]. Both antidepressant-like effects and increase of hippocampal histone H4 acetylation have been observed after repeated administration of sodium butyrate [199], along with normalization of hippocampal BDNF expression, histone H3 acetylation and decrease of chronic restraint stress-induced depressive behaviors [200]. Remarkably, not only butyrate administration show antidepressant potential but one of the recognized butyrate-producer bacterial species, *F. prausnitzii*, has showed to exert comparable antidepressant effects against chronic unpredictable stress (CUS)-induced depression-like behavior in rats [201]. Moreover, *F. prausnitzii* administration reestablishes an anti-inflammatory environment by increasing plasma levels of interleukin-10 (IL-10) and preventing the increase of stress-induced release of pro-inflammatory C-reactive protein and interleukin-6 (IL-6) [201]. In addition to the well-established correlation between antidepressant therapy and increase of BDNF expression in hippocampus and PFC of depressed patients [202], monoamine deficiency is regarded as the main explicative hypothesis of depression pathophysiology and, consequently, SSRIs, SNRIs and MAO inhibitors the major remedial therapeutics. A reciprocal regulation seem to exist between 5-HT transmission and BDNF expression, whereby BDNF contribute to 5-HT neurons differentiation, development and function and, on the other hand, potentiation of 5-HT signaling (e.g., via SSRIs administration) promotes neural and astrocyte BDNF expression [203]. The antidepressant effects of butyrate administration seem to involve the same mutual regulation between BDNF expression and 5-HT neurotransmission. Hence, several HDAC inhibitors (including butyrate) were shown to promote cell differentiation via the potentiation of 5-HT-induced BDNF gene expression [204], and butyrate administration was shown to counteract CUS-induced anhedonic symptoms via the increase of 5-HT brain levels and reversal of CUS-induced decrease of BDNF expression [205]. The fact that SCFAs are involved in neuroplasticity, neurogenesis, consolidation of long-term memory, and in maintenance of BBB integrity [197,198,206,207], provides further mechanistic support to the idea that butyrate and bacterial-producing SCFAs may be promising dietary-derived neuroprotective and antidepressant agents. Of note, not only butyrate but also propionate can exert a protective action against microbial infection and oxidative stress-induced increase of BBB permeability [208]. Indeed, the ability of SCFAs to confer protection against derangement of MBC and defective BBB integrity is of foremost importance for the preservation of the major defensive structure of the brain and the fight against NPDs pathogenesis. Finally, changes of bacterial composition and increase in valeric acid production has been described in positive correlation with depression symptoms [209], a SCFA that can affect neurotransmitter release as for instance acting on glycine or adenosine receptors [210,211], which antagonism may have synergistic antidepressant effects [212].

#### 4.2.2. SCFAs, Dysbiosis and Neuroinflammation in ASD and SCZ

The pathophysiological connection between ASD and alterations of gut microbial community sounds, at first sight, quite surprisingly. Nevertheless, such connection does exist and possibly represents the most concrete example of the consequences of defective MBC for the pathogenesis of NPDs. ASD is an elusive, and somehow, enigmatic neurodevelopmental syndrome, affecting deeply multiple aspects of behavior (social interaction, motor stereotypies, self-injury) and intensely the realm of communication. Essentially, the pathophysiological relationship between MBC and ASD is supported by the significant amount of GI disorders in ASD subjects. Moreover, further evidence comes from the correlation between severity of the ASD clinical signs and the accentuation of GI symptoms such as abdominal pain, bloating and constipation and/or diarrhea [213,214,215]. Among the first hypotheses of comorbidity between ASD and GI disorders, it might be mentioned a seminal paper [216] in which low-grade intestinal inflammation (i.e., induced by *Clostridium tetani*) was considered to play an etiological role in ASD pathogenesis. Later, several studies have confirmed the existence of lower *Bacteroides* to *Firmicutes* ratio and increase of *Clostridiales* in autistic children [217,218], and pyrosequencing analysis contributed to identify the bacterial genus *Desulfovibrio* as more highly represented in autistics than in non-autistic subjects [219]. Interestingly, the Gram-negative anaerobic bacterium *Desulfovibrio* can generate lipopolysaccharide (LPS), thus supporting the concept of low-grade endotoxemia ASD patients [220]. In general, the *Proteobacteria* phylum is overrepresented in children with ASD, especially in those with mental retardation [219,221], and its spreading is often associated with IBS, gut inflammation and LPS production [219,222]. LPS-induced endotoxemia triggers alteration of social behavior in the offspring, even in case of prenatal immune challenge [223,224]. Moreover, LPS serum levels have been found higher in ASD patients [220]. Interestingly, *Desulfovibrio* species are sulfate-reducing bacteria and sulfur amino acid (SAA) metabolism appears defective in autistic subjects. Indeed, reduced sulfur plasma levels and abnormal sulfur urinary excretion may in part account for the alteration of immune function in ASD [225,226]. Concerning dietary interventions, the probiotic supplementation with a pool of different strains of *Lactobacillus*, *Bifidobacterium* and *Streptococcus* has been reported to reduce the *Bacteroides* to *Firmicutes* ratio and the prevalence of *Desulfovibrio* genus [218]. Moreover, beyond the dramatic bacteria alterations in the gut of children with ASD, other gut-derived metabolites such as the free amino acids (FAA) resulting from proteins and peptides hydrolysis, have been found associated with ASD and higher in autistic individuals [227]. Among the multiple animal models of ASD, it has been observed that the supplementation with *Bacteroides fragilis* in the offspring generated by the model of maternal immune activation (MIA) significantly re-equilibrated microbial composition, reduced gut permeability and ASD-like behaviors such as social communication and anxiety [228]. On the other hand, SCFAs may have quite different effects in ASD pathogenesis. Higher than normal levels of PPA, BA and valeric acid have been reported in autistic subjects [229]. Such abnormal levels may be, at least in part, be attributed to the unbalance towards specific predominant bacterial populations in ASD as for the mentioned *Clostridia*, *Bacteroides* and *Desulfovibrio*, which are all key SCFA producers, and in particular of PPA [230,231]. Thus, while physiological levels of PPA are involved in modulation of immune function, gene expression and mitochondrial and lipid metabolism [232,233,234,235], the abnormal production/increase of PPA promotes neuroinflammation by the release of pro-inflammatory cytokines and gliosis by excessive proliferation of glial progenitor cells and derangement of the neuron/glia ratio as reported in ASD patients [236]. Of note, exposure to PPA in juvenile and adult rats has been developed as a model of autism to reproduce ASD-like brain alterations (e.g., neuroinflammation and oxidative stress) and dysfunctional behaviors such as repetitive dystonic movements, hyperactivity and deficit of social interaction. [237]. Abnormal PPA blood accumulation is also observed in the clinical condition known as propionic acidemia (PA), in which the fault of catabolism of branched-chain amino acids (namely, the activity of the enzyme propionyl-CoA carboxylase, PCC) leads to the mitochondrial accumulation of propionyl-CoA and mitochondrial dysfunction. Impaired mitochondria function is similarly observed in subjects with autism as well as in animals subjected to i.c.v. PPA exposure [233,238]. Although dietary factors may have a key role in shaping gut microbiota ecosystem, our knowledge about the possible dietary interventions to modify gut bacterial phylotypes in ASD subjects is still inadequate. Interestingly, a recent study did not found significant association between dietary patterns, fecal microbiota composition and changes in severity of social deficit in ASD children [239]. Nevertheless, in the same study, the intake of specific nutrients and consumption of specific healthy or unhealthy dietary patterns, was found able to modulate the major incidence of selected either beneficial or deleterious bacteria taxa and SCFAs production. Amongst the different dietary interventions or targeted nutritional approaches that have been suggested as potential therapies in ASD, the gluten-free/casein-free (GF/CF) diet, the ketogenic diet and probiotic supplementation have been intensely investigated in the last years [240].

Similarly to children with ASD, also in other NPDs such as schizophrenia (SCZ) and bipolar disorder (BD) there is a marked alteration of gut microbiota populations in comparison to healthy subjects. A greater abundance of bacteria from the *Lactobacillus* group was described in a study focused on patients with first-episode psychosis, which was also found to correlate with severity of positive symptoms [241]. Moreover, in the same study, the overrepresentation of *Lachnospiraceae* and *Ruminococcaceae* families was found to correlate with severity of negative symptoms [241]. According to a later study [242], SCZ patients show reduced microbial diversity of the gut flora with an increased incidence of *Lachnospiraceae*, *Bacteroidaceae* and *Streptococcaceae* microbiota species and in linear correlation with symptoms severity. Since prebiotics supplementation can increase BDNF levels and probiotics reestablish hippocampal BDNF expression after social stress [38,243], the relationship between SCFAs production and BDNF function may have a major critical importance for the implication of gut microbiota in SCZ pathogenesis. The strong involvement of BDNF in SCZ [244,245] stems from the multiple functions of this neurotrophin such as its importance for brain development, neural differentiation, neurotransmitter release, neuronal plasticity, cognitive changes, protection and survival of DAergic, 5HT and cholinergic neurons [246,247]. Antibiotic treatment or GF mice show altered BDNF expression in several brain regions involved in SCZ, including hippocampus and cingulate cortex [17,248]. As observed for the role of SCFAs in depression, butyrate can normalize BDNF expression and depression-like behaviors in animals [200], through mechanisms involving BDNF-5HT synergistic modulation as well as HDAC inhibition and potentiation of 5HT transmission [204]. Not only butyrate administration can promote the recovery of BDNF expression and memory impairment [249], but its activity as HDAC inhibitor provides a mechanistic evidence for the ability to suppress several LPS-induced pro-inflammatory factors [250], which are recognized components of SCZ pathogenesis [251]. Strikingly, stress-induced derangement of gut microbial composition and changes in brain BDNF expression are associated to the alteration of NMDA receptor subunits, such as for the decrease of GluN2A subunit in the hippocampus and cortex of GF mice [32]. The hypofunction of NMDA receptor is regarded as one influential hypothesis in SCZ pathophysiology [252], and sporadic mutations of the *GRIN2A* gene encoding the GluN2A subunit have been described in both SCZ and ASD patients [253]. Prebiotics supplementation, in the form of fructo-oligosaccharides (FOS) and galacto-oligosaccharides (GOS), not only promotes hippocampal BDNF increase but also increased the expression of hippocampal GluN2A subunit, thus providing additional evidence that prebiotics-dependent *Bifidobacteria* proliferation facilitates the expression of some NMDA receptor subunits [243]. Moreover, fecal microbiome transplantation from SCZ patients to GF mice produced abnormal hypothalamic GABA and glutamine increase, concomitant glutamatergic hypofunction and SCZ-like behaviors [242]. Concerning BD, a recent comparative analysis of the stool microbiome of patients with BD has highlighted a major decrease of the phylum *Firmicutes*, and in particular of the BA-producing *Faecalibacterium* [169,254], whose administration has demonstrated potential antidepressant-like effects [201], and whose deficiency in gut microbiota is considered a marker of several inflammatory clinical conditions such as CD [185].

## 5. Conclusive Remarks

The landscape of gut microbiota can be radically shaped by dietary fats, and even in opposite direction by lipids of different types. Indeed, the present discussion has provided broad evidence that dietary lipids such as SFAs can determine dysbiosis and liability to NPDs, and that selected lipids (i.e., *n*-3 PUFAs) and their metabolites are able to provide disease resilience or “resolve” the underlying systemic and brain inflammation involved in SCZ, ASD and depression pathogenesis. While robust evidence supports the view that abnormal increase of *n*-6 to *n*-3 ratio is a major pathogenetic link between dietary lipids, derangement of microbial ecosystem and increased risk of NPDs (see in particular Section 3.2 and Section 3.4 and Figure 2), there are also data disclosing an intriguing connection between n-3 PUFAs levels, microbiota diversity and SCFAs production [255]. In a recent population-based study, higher circulating levels of DHA were found to positively correlate with higher microbiome diversity and higher abundance of *Lachnospiraceae* bacterial family, irrespective of dietary fibre intake. Considering the *Lachnospiraceae* family as one important SCFAs “producer” this study suggests a potential additional mechanism underlying the link between n-3 PUFAs levels, gut microbiota health and lower risk of NPDs. On the same lines of evidence, depression-like behaviors elicited in mice by social isolation were found not only associated with a shift in microbiota composition but also with a decrease of SCFAs-producing bacteria (e.g., *Allobaculum*), which was sensitive to DHA dietary intervention [256].

The present survey has shown extensive evidence of the multiple associations connecting the unbalanced consumption of selected dietary fatty acids to the risk of NPDs. By extending the current knowledge to the mechanistic link between dietary lipids, deranged microbial population and changes in neuroactive compounds (particularly DA and 5-HT), we may sensibly improve our information about NPDs pathogenesis and design novel interventional strategies. Developing a wider understanding of these mechanisms may contribute to define innovative guidelines of prevention by dietary intervention, as well as to improve the identification of microbiota- and NPDs-associated biomarkers to potentiate both early diagnosis and personalized medicine.
